# Supplementary material for: Mutations in PCYT2 disrupt etherlipid biosynthesis and cause a complex hereditary spastic paraplegia
Source: Brain. 2019 Oct 22;142(11):3382–97. doi: 10.1093/brain/awz291 (PMC6821184; doi:10.1093/brain/awz291)
Supplement: awz291_Supplementary_Data [file awz291_supplementary_data.zip › awz291-Suppl_data/Supplementary_Data4.zip!/Supplementary information 3 - fibroblast lipidomics.zip!/Fibroblast lipidomics/Controle vs Patient2/Statistical plots and data/CL species/Heatmap.pdf]

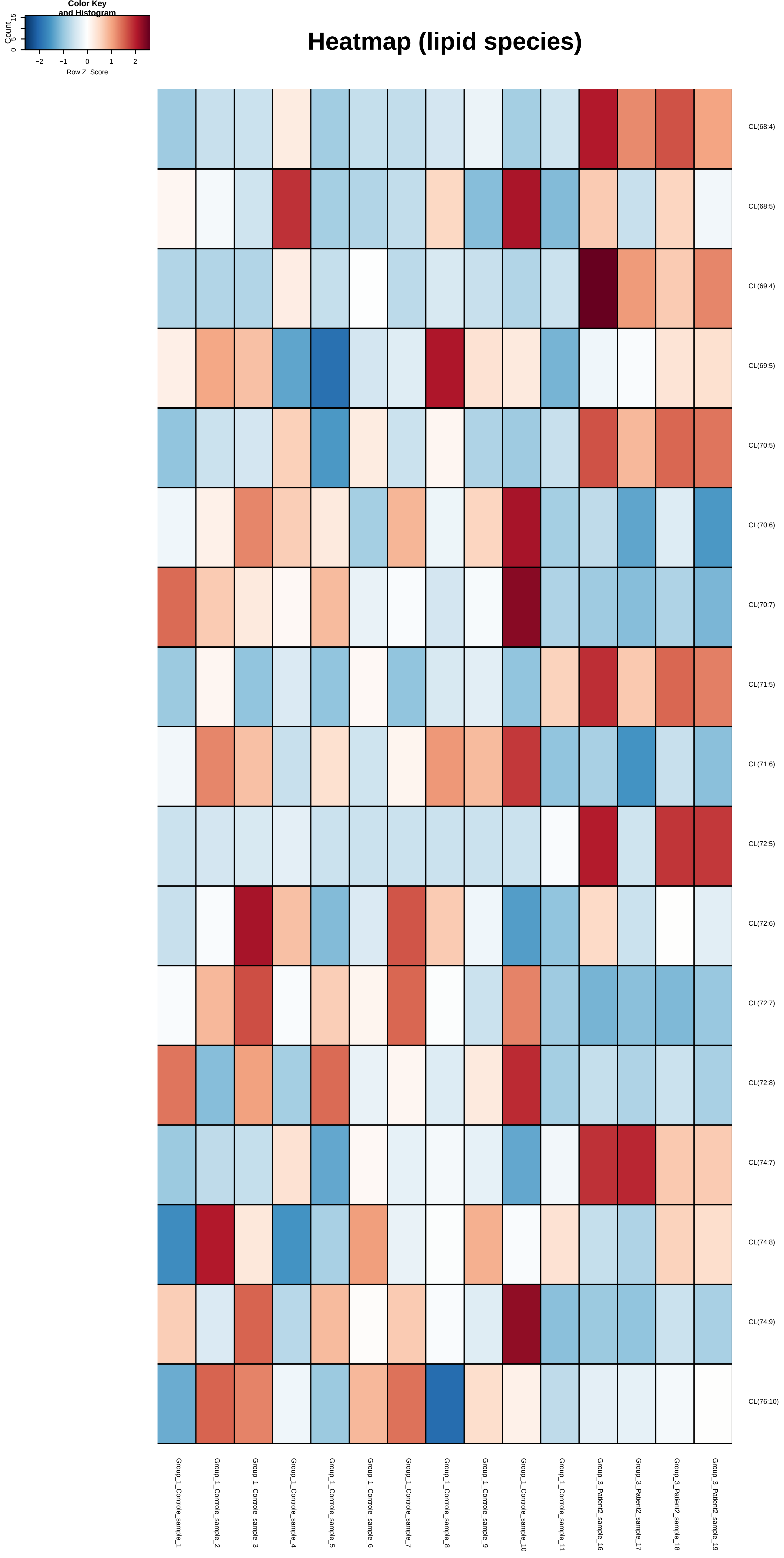
Color Key and Histogram
Count
0
5
15
−2
−1
0
1
2
Row Z−Score
Heatmap (lipid species)
CL(68:4)
CL(68:5)
CL(69:4)
CL(69:5)
CL(70:5)
CL(70:6)
CL(70:7)
CL(71:5)
CL(71:6)
CL(72:5)
CL(72:6)
CL(72:7)
CL(72:8)
CL(74:7)
CL(74:8)
CL(74:9)
CL(76:10)
Group_1_Controle_sample_1
Group_1_Controle_sample_2
Group_1_Controle_sample_3
Group_1_Controle_sample_4
Group_1_Controle_sample_5
Group_1_Controle_sample_6
Group_1_Controle_sample_7
Group_1_Controle_sample_8
Group_1_Controle_sample_9
Group_1_Controle_sample_10
Group_1_Controle_sample_11
Group_3_Patient2_sample_16
Group_3_Patient2_sample_17
Group_3_Patient2_sample_18
Group_3_Patient2_sample_19
